# Supplementary material for: Primordial germ cell DNA demethylation and development require DNA translesion synthesis
Source: Nat Commun. 2024 May 3;15:3734. doi: 10.1038/s41467-024-47219-2 (PMC11068800; doi:10.1038/s41467-024-47219-2)
Supplement: Supplementary file 1 — Supplementary Information [file 41467_2024_47219_MOESM1_ESM.pdf]

Supplementary information for:

Primordial germ cell DNA demethylation and development require DNA translesion synthesis.

Pranay Shah[1*], Ross Hill[1], Camille Dion[2,3], Stephen J. Clark[4,5], Jeroen Willems[6], Abdulkadir Abakir[4,5], Mark J. Arends[7], Wolf Reik[4,5], Harry G. Leitch[2,3], Juan I Garaycoechea[6], Gerry P. Crossan[1*]

Affiliations

1. MRC Laboratory of Molecular Biology, Cambridge, UK
2. MRC Laboratory of Medical Sciences, London, W12 0HS, UK
3. Institute of Clinical Sciences, Faculty of Medicine, Imperial College London, London, W12 0HS, UK
4. Altos Labs, Cambridge, UK
5. Epigenetics Programme, The Babraham Institute, Cambridge CB22 3AT, UK
6. Hubrecht Institute, Royal Netherlands Academy of Arts and Sciences (KNAW), Utrecht, the Netherlands
7. Cancer Research UK Edinburgh Centre, Edinburgh, UK

* Corresponding authors: gcrossan@mrc-lmb.cam.ac.uk or pshah@mrc-lmb.cam.ac.uk

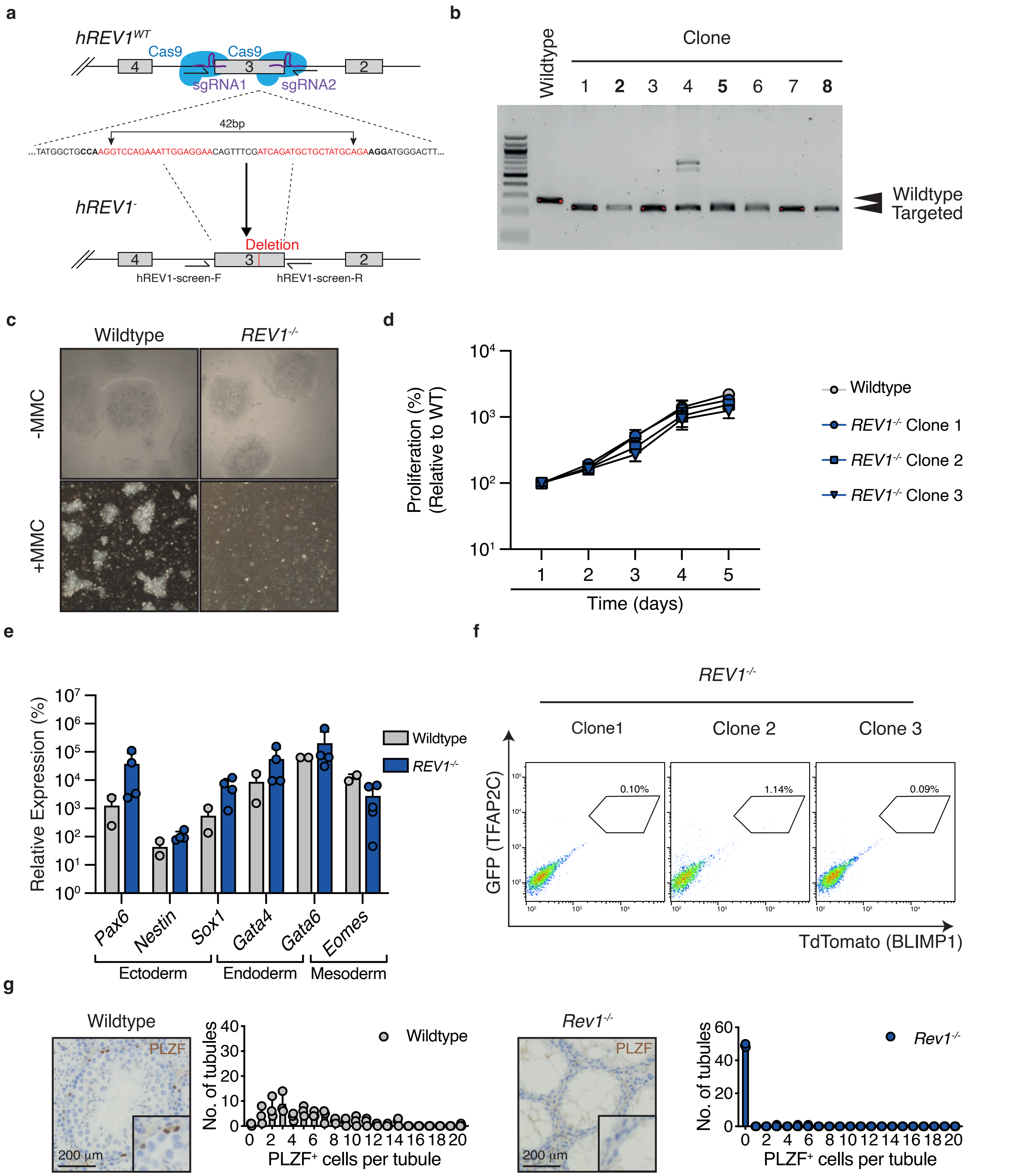

Supplementary Figure 1. Generation of REV1-deficient hiPSCs and characterization of *Rev1*$^{-/-}$ mouse testes.

Supplementary Figure Legends:

Supplementary Figure 1. Generation of REV1-deficient hiPSCs and characterization of *Rev1*$^{-/-}$ mouse testes.

(a) Schematic representation of the *REV1*$^{-/-}$ allele in hiPSCs and targeting of the *REV1* locus with gRNAs.

(b) Gel image of *REV1* PCR amplicons from single cell derived colonies. Size of the wildtype amplicon is 281bp and of the targeted amplicon is 239bp.

(c) Representative brightfield images of untreated and mitomycin c (MMC) treated wildtype and *REV1*$^{-/-}$ hiPSCs.

(d) Proliferation of wildtype and 3 *REV1*$^{-/-}$ hiPSC clones measured over 5 days. Data represent mean and s.d.

(e) Analysis of the expression of genes from the three germ layers in day 21 embryoid bodies generated from *REV1*$^{-/-}$ and parental wildtype hiPSCs by RT-qPCR(n= 2 for wildtype and 4, 4, 4, 4 and 5 for *REV1*$^{-/-}$ left to right). Data represent mean.

(f) Representative flow cytometry plots of *TFAPC*-GFP and *BLIMP1*-tdTomato positive hPGCLCs at day 4 of aggregate differentiation from from *REV1*$^{-/-}$ clones.

(g) Images of PLZF-stained testes and distribution of PLZF^{+} cells per seminiferous from 8–12-week-old wildtype (left) and *Rev1*$^{-/-}$ (right) mice (n=150 tubules per genotype, 50 per mouse). Data represent mean and s.d.

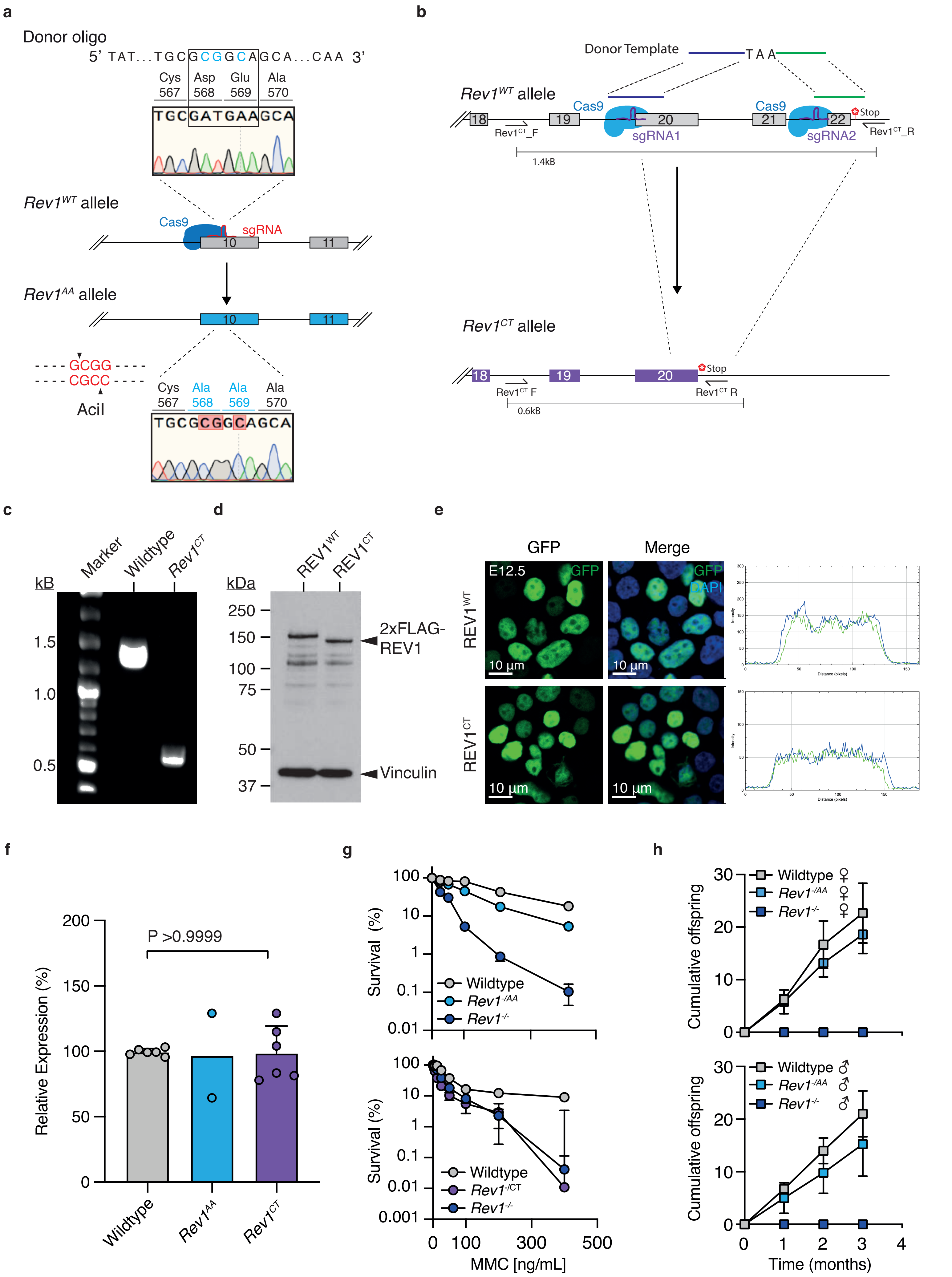

Supplementary Figure 2. Generation and validation of Rev1AA and Rev1CT alleles by CRISPR-Cas9 genome editing.

Supplementary Figure 2. Generation and validation of Rev1AA and Rev1CT alleles by CRISPR-Cas9 genome editing

(a) Generation of the *Rev1*^{AA} allele in mouse embryonic stem cells (mESCs). Sanger sequencing profiles of the wildtype (top) and targeted *Rev1*^{AA} (bottom) locus from confirming successful mutagenesis of the catalytic residues D568 and E569 to alanines (D568A and E569A).

(b) Generation of the *Rev1*^{CT} allele by CRISPR-Cas9 genome editing in mouse zygotes. Two sgRNAs (sgRNA1 and sgRNA2) were used to target Cas9 activity to sites either side of REV1's C-terminus. A template DNA molecule (donor template) was then used to insert a stop codon (TAA) and truncate the C-terminus.

(c) Verification of gene targeting by long-range PCR. Oligonucleotide pairs were designed either side of the DNA encoding the C-terminal 100 amino acids of REV1. In the wildtype *Rev1* allele a 1,500 bp band is amplified. This is truncated to a 600 bp product in the *Rev1*^{CT} allele.

(d) Anti-FLAG immunoblot from cells expressing N-terminally FLAG-tagged full length and C-terminally truncated REV1.

(e) Left: Representative immunofluorescence images of cells expressing GFP-tagged full length and C-terminally truncated REV1. Right: Representative GFP and DAPI intensity plots from GFP^{+} cells.

(f) RT-qPCR of the *Rev1* transcript in wildtype, *Rev1*$^{-/AA}$ and *Rev1*$^{-/CT}$ mouse embryonic fibroblasts (n = 6, 2 and 6, left to right). Data represent mean and s.d. P value calculated by two-tailed Mann-Whitney *U*-test

(g) Sensitivity of wildtype, *Rev1*$^{-/-}$ and *Rev1*$^{-/AA}$ (top) or *Rev1*$^{-/CT}$ (bottom) cells to MMC (data represent 3 independent experiments each carried out in triplicate). Data represent mean and s.d.

(h) Cumulative number of offspring when mutant female (top) or male (bottom) mice were mated with wildtype mates and checked for evidence of copulation (n=3 independent mice per genotype and sex). Data represent mean and s.d.

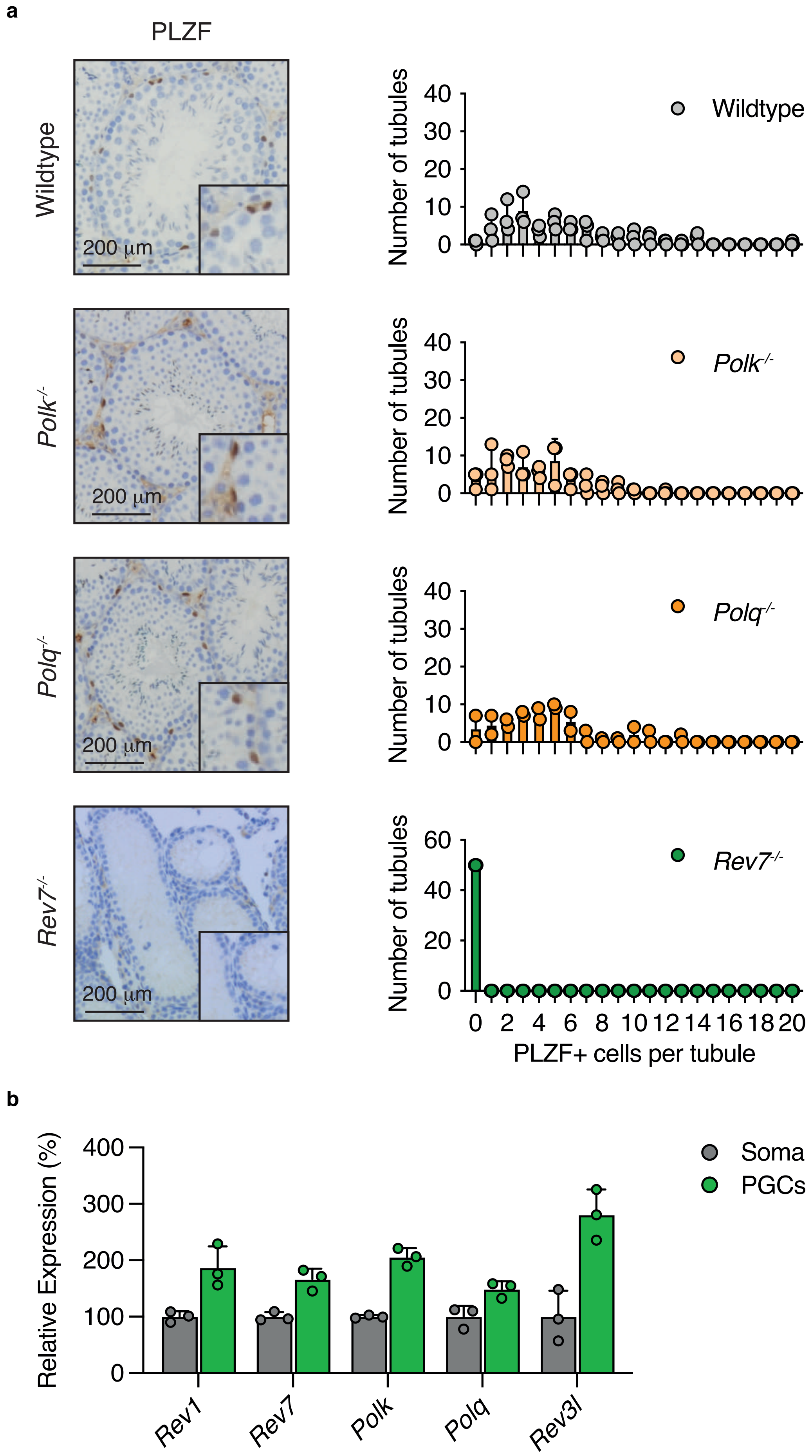

Supplementary Figure 3. Lack of PLZF+ cells in the testes of *REV7*-deficient mice.

Supplementary Figure 3. Lack of PLZF$^+$ cells in the testes of REV7-deficient mice

(a) Representative images of testis sections stained for PLZF and the quantification of PLZF$^+$ cells per seminiferous tubule of wildtype, *Polk*$^{-/-}$, *Polq*$^{-/-}$ and *Rev7*$^{-/-}$ mice (wildtype, *Polk*$^{-/-}$ and *Rev7*$^{-/-}$ n=150 tubules per genotype, 50 per animal, *Polq*$^{-/-}$ n=100 tubules, 50 per animal). Data represent mean and s.d.

(b) Droplet digital PCR (ddPCR) gene expression analysis of *Rev1*, *Rev7, Polk*, *Polq* and *Rev3l* in FACS-purified PGCs and surrounding somatic cells (SSEA1$^-$GOF18-GFP$^-$) from E10.5 embryos (n=3 independent embryos). Data represent mean and s.d.

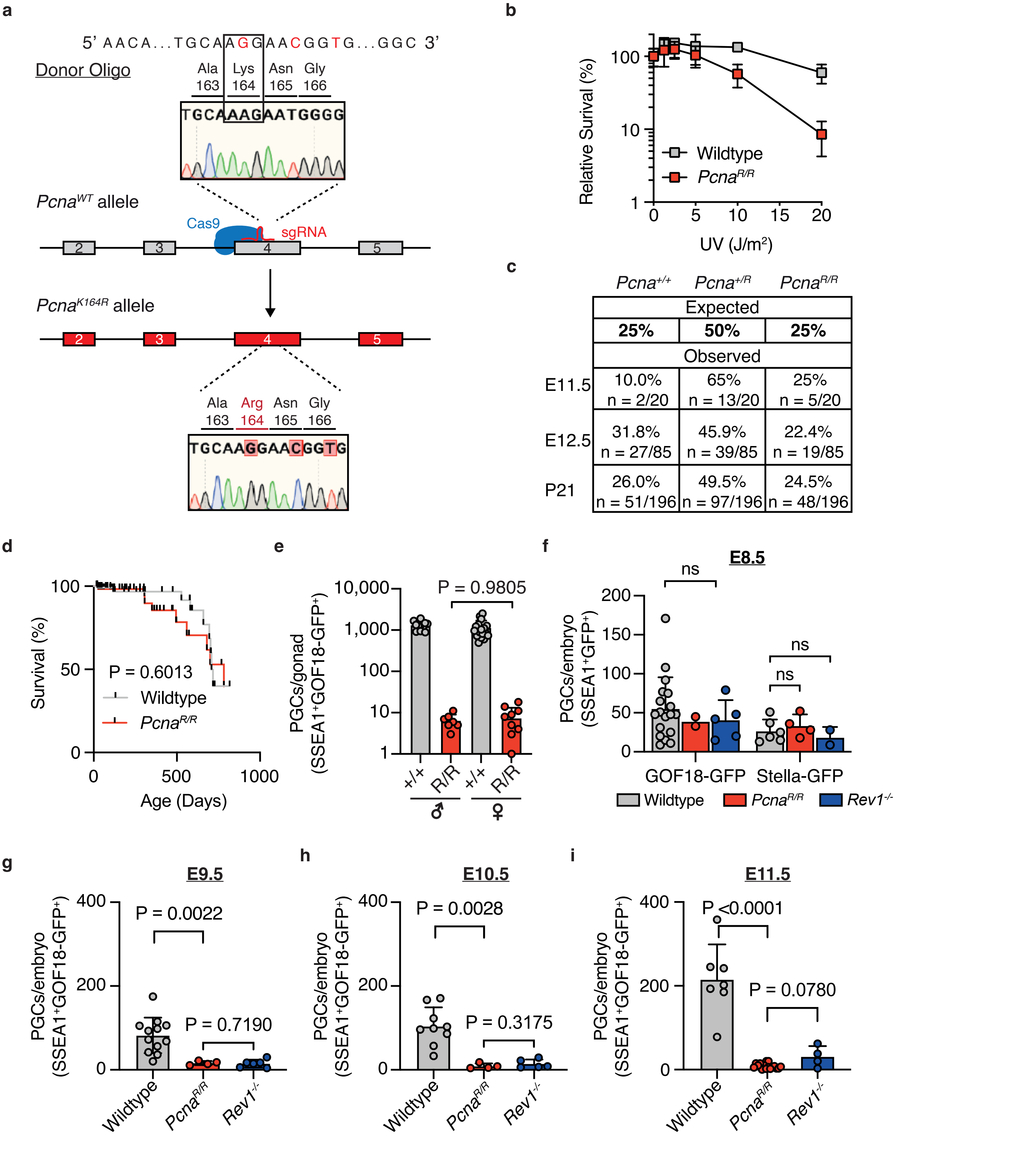

Supplementary Figure 4. Generation and characterisation of *Pcna*$^{R/R}$ mice.

Supplementary Figure 4. Generation and characterisation of *Pcna*$^{R/R}$ mice

(a) Generation of the *Pcna*^{K164R} allele by CRISPR-Cas9 genome editing in mouse zygotes. Sanger sequencing profiles of the wildtype (top) and targeted (bottom) *Pcna* locus confirming successful mutagenesis.

(b) Sensitivity of wildtype and *Pcna*$^{R/R}$ MEFs to ultraviolet (*57*) irradiation (data represent 3 independent experiments each carried out in triplicate).

(c) *Pcna*$^{R/R}$ embryos are observed at the expected Mendelian ratios throughout development (E11.5 and E12.5) and at 21 days postpartum (P21).

(d) Kaplan-Meier survival curve for a cohort of wildtype and *Pcna*$^{R/R}$ mice (n=125 wildtype mice, 62 *Pcna*$^{R/R}$ mice, P values were calculated by the Mantel-Cox test).

(e) Quantification of PGCs by flow cytometry from male and female embryos at E12.5 (Data represent mean and s.d., P value calculated by two-tailed Mann-Whitney *U*-test; *n*=13, 17, 7 and 10 independent embryos, left to right).

(f) Quantification of PGCs by flow cytometry from wildtype, *Rev1*$^{-/-}$ and *Pcna*$^{R/R}$ embryos at E8.5 (Data represent mean and s.d., P value calculated by two-tailed Mann-Whitney *U*-test; wildtype, n = 18 and 6; *Rev1*$^{-/-}$, n = 5 and 2; *Pcna*$^{R/R}$, n = 2 and 4, independent embryos, left to right).

(g-i) Quantification of PGCs by flow cytometry through development (E9.5-11.5) from wildtype, *Pcna*$^{R/R}$ and *Rev1*$^{-/-}$ embryos (Data represent mean and s.d., P value calculated by two-tailed Mann-Whitney *U*-test; E9.5, n=12, 4 and 6; E10.5, n=9, 4 and 5; E11.5, n=7, 16 and 4; left to right).

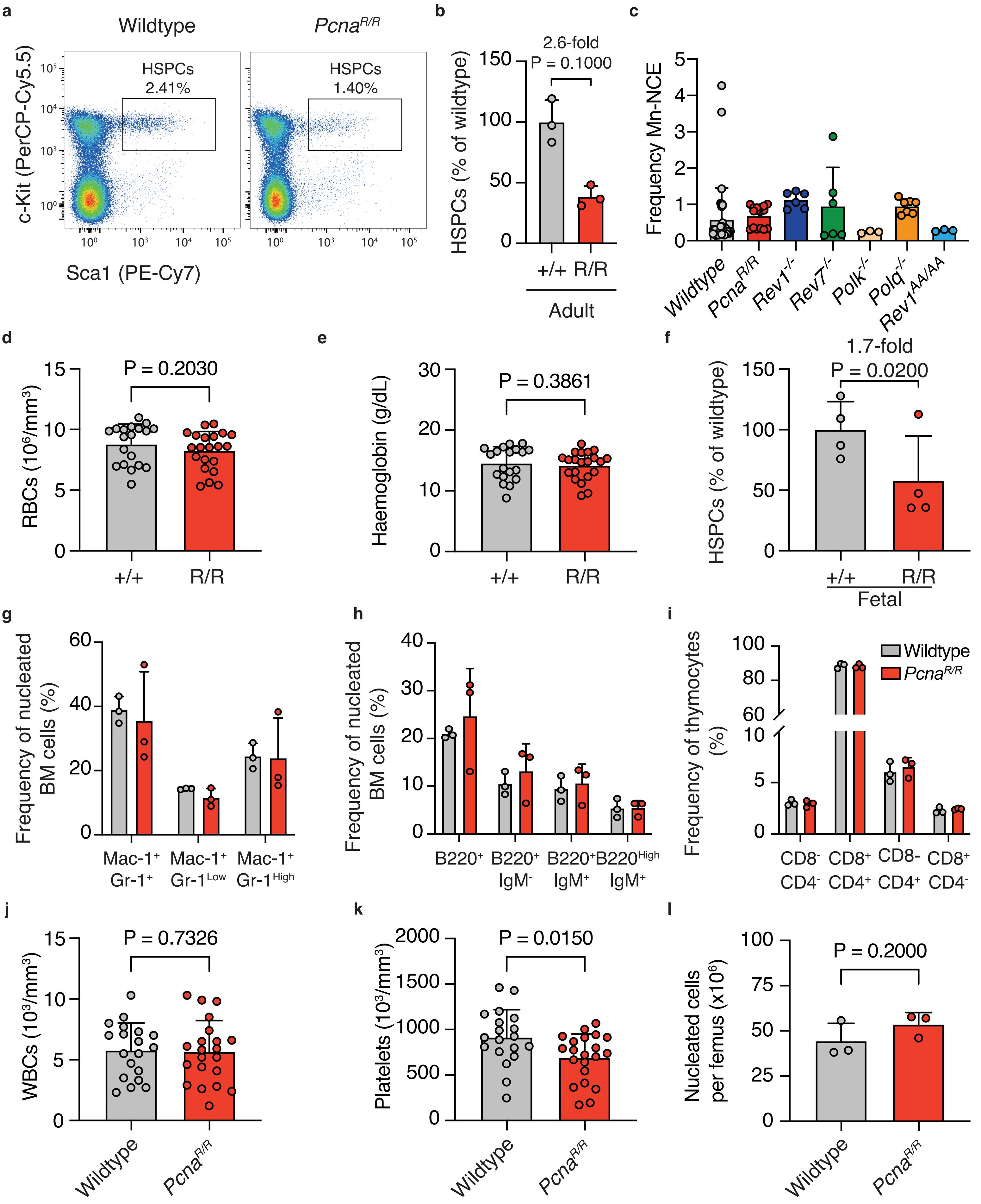

Supplementary Figure 5. Normal haematopoiesis in *Pcna*$^{R/R}$ mice.

Supplementary Figure 5. Normal haematopoiesis in *Pcna*$^{R/R}$ mice

(a) Representative flow cytometry plots of hematopoietic stem and progenitor cells (HSPCs) of wildtype and *Pcna*$^{R/R}$ mice.
(b) Quantification of HSPCs (Lin-Kit^{+}Sca-1^{+}) by flow cytometry from 8-12-week-old wildtype and *Pcna*$^{R/R}$ mice (Data represent mean and s.d., P value calculated by two-tailed Mann-Whitney *U*-test; n=3 independent mice per genotype).
(c) Frequency of micronucleated normochromic erythrocytes (Mn-NCE) quantified by flow-cytometry (Data represent mean and s.d., n = 37, 16, 6, 6, 3, 7 and 3 left to right; P value calculated by two-tailed Mann-Whitney *U*-test: Wildtype vs *Pcna*$^{R/R}$ P=0.0048; Wildtype vs *Rev1*$^{-/-}$ P=0.0019; Wildtype vs *Rev7*$^{-/-}$ P=0.9114; Wildtype vs *Polk*$^{-/-}$ P=0.5984; Wildtype vs *Polq*$^{-/-}$ P=0.0015; Wildtype vs *Rev1*$^{AA/AA}$ P=0.8358.)
(d) Red blood cell (RBC) counts of 8-12-week-old wildtype and *Pcna*$^{R/R}$ mice (Data represent mean and s.d., P value calculated by two-tailed Mann-Whitney *U*-test; n=18 wildtype and 21 *Pcna*$^{R/R}$ mice).
(e) Blood haemoglobin concentration quantification of 8-12-week-old wildtype and *Pcna*$^{R/R}$ mice (Data represent mean and s.d., P value calculated by two-tailed Mann-Whitney *U*-test ; n=18 wildtype and 21 *Pcna*$^{R/R}$ mice; RBC, red blood cells; WBC, white blood cells).
(f) Quantification HSPCs (Lin-Kit^{+}Sca-1^{+}) by flow cytometry from wildtype and *Pcna*$^{R/R}$ embryos at E12.5 (Data represent mean and s.d., P value calculated by two-tailed Mann-Whitney *U*-test ; n=4 independent mice per genotype).
(g) Quantification of myeloid cell by flow cytometry from bone marrow of 8-12-week-old wildtype and *Pcna*$^{R/R}$ mice stained for the myeloid markers Mac-1 and Gr-1 and the quantification of distinct populations (Data represent mean and s.d.; n=3 independent mice per genotype).
(h) Quantification of B-cell maturation by flow cytometry from bone marrow of 8-12-week-old wildtype and *Pcna*$^{R/R}$ mice stained for the B cell markers B220 and IgM and the quantification of distinct populations (Data represent mean and s.d.; n=3 independent mice per genotype).
(i) Quantification of T-cell development by flow cytometry from the thymus of 8-12-week-old wildtype and *Pcna*$^{R/R}$ mice stained for the T cell markers CD4 and CD8 and the quantification of distinct populations (Data represent mean and s.d.; n=3 independent mice per genotype).

(j-l) Whole blood counts and quantification of nucleated cells per femurs of 8-12-week-old wildtype and *Pcna*$^{R/R}$ mice (Data represent mean and s.d., P value calculated by two-tailed Mann-Whitney *U*-test; n=18 wildtype and 21 *Pcna*$^{R/R}$ mice; WBC, white blood cells).

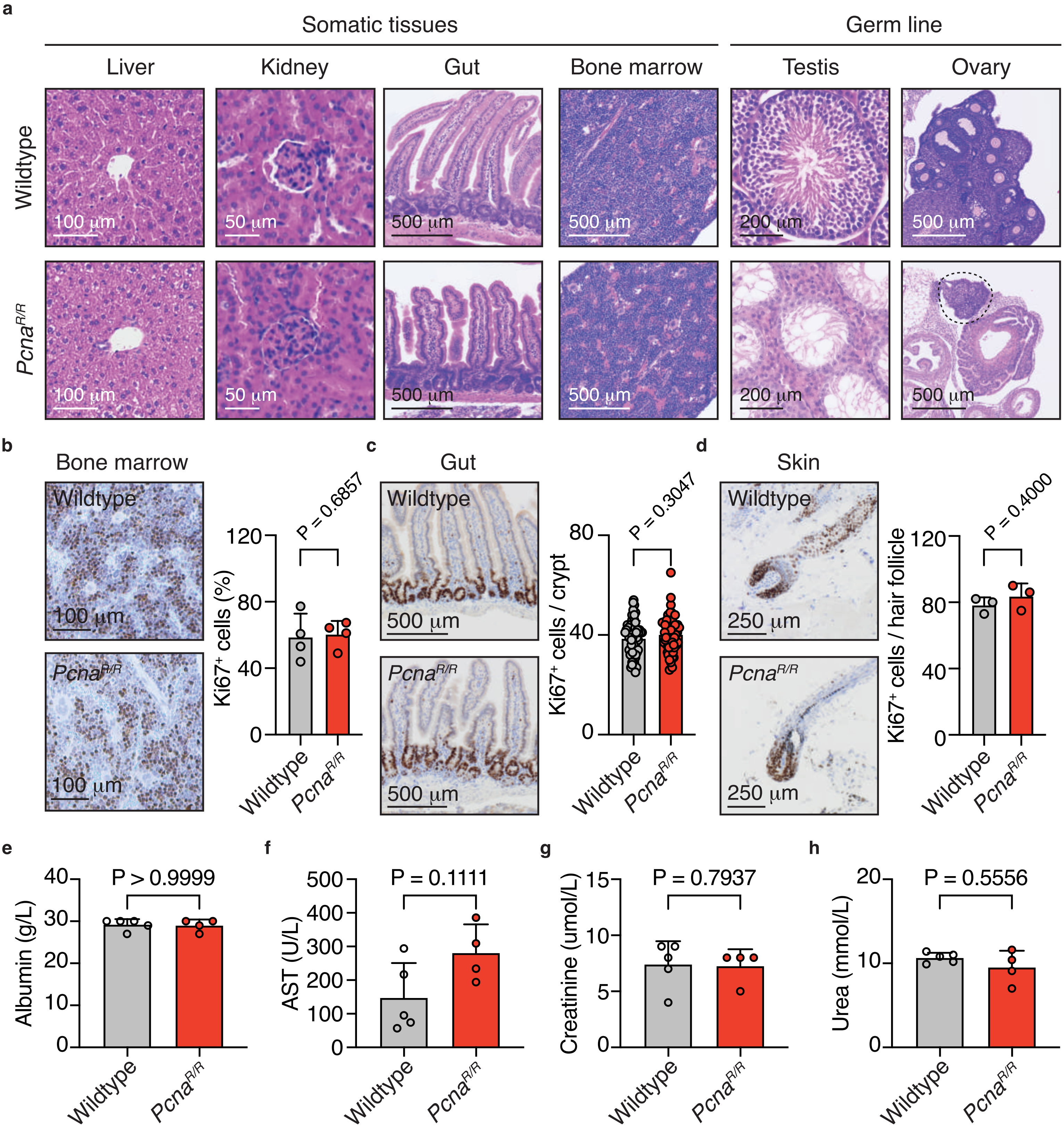

Supplementary Figure 6. Analysis of somatic tissues from *Pcna*$^{R/R}$ mice.

Supplementary Figure 6. Analysis of somatic tissues from *Pcna*$^{R/R}$ mice

(a) H&E-stained sections of liver, kidney, gut and bone marrow sections (left) and testis and ovary sections (right) from wildtype and *Pcna*$^{R/R}$ adult mice (similar observations were made in 3 independent animals per genotype).

(b) Representative images of bone marrow sections from 8-12-week-old wildtype and *Pcna*$^{R/R}$ mice stained for Ki67 and quantification of the frequency of Ki67^{+} cells (Data represent mean and s.d., P value calculated by two-tailed Mann-Whitney *U*-test; n=3 independent mice per genotype).

(c) Representative images of ileum sections stained for Ki67 from 8-12-week-old wildtype and *Pcna*$^{R/R}$ mice and quantification of Ki67^{+} cells per crypt of the ileum (Data represent mean and s.d., P value calculated by two-tailed Mann-Whitney *U*-test; n=60 crypts per genotype, with 20 per mouse per genotype).

(d) Representative images of skin sections from 8-12-week-old wildtype and *Pcna*$^{R/R}$ mice stained for Ki67 and quantification of Ki67^{+} cells per hair follicle bulge (Data represent mean and s.d., P value calculated by two-tailed Mann-Whitney *U*-test; n=3 independent mice per genotype).

(e-h) Serum levels of albumin, AST, creatinine and urea (Data represent mean and s.d., P value calculated by two-tailed Mann-Whitney *U*-test; wildtype n=5;*Pcna*$^{R/R}$ n=4).

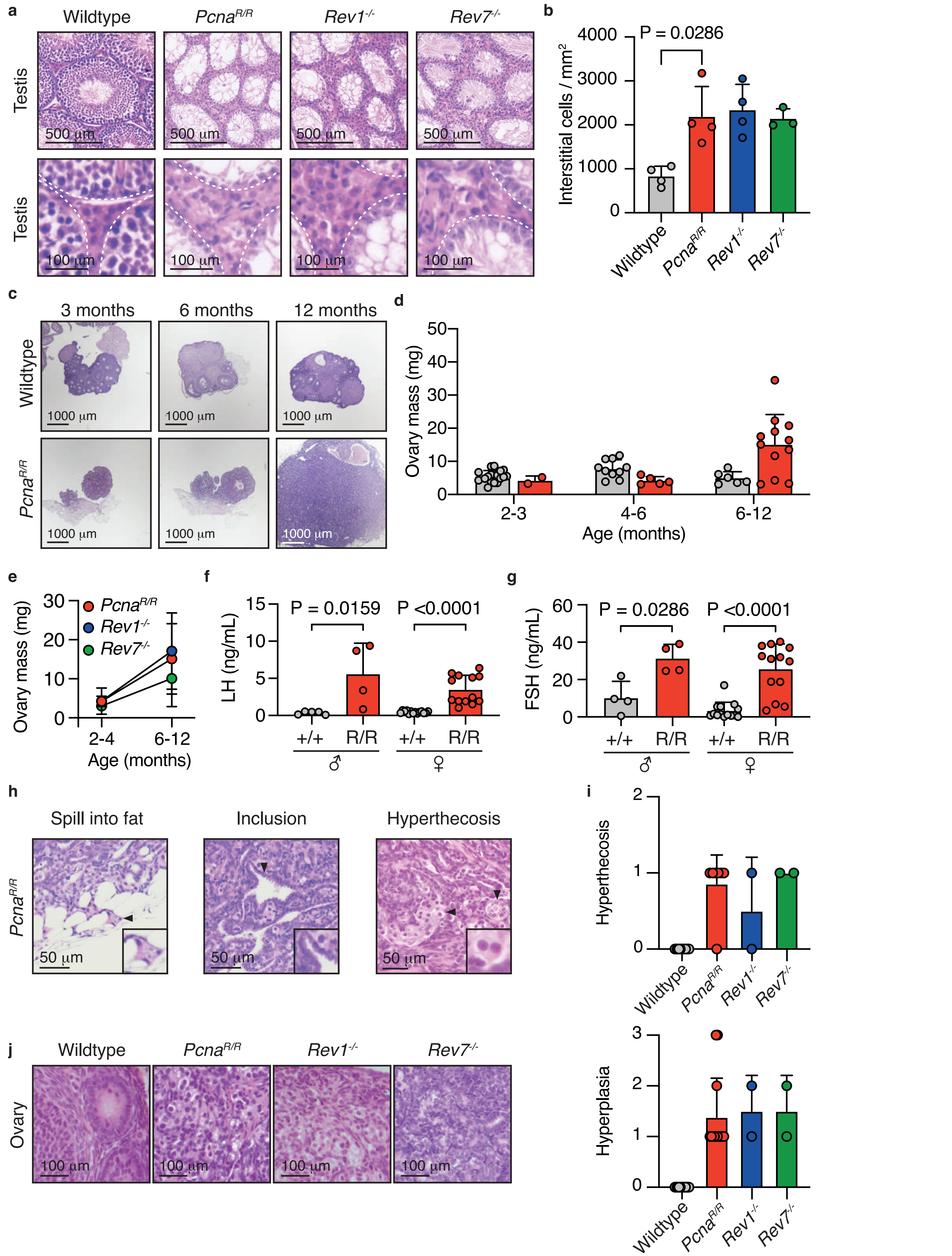

Supplementary Figure 7. The hypothalamic-pituitary-gonadotrophic axis is disrupted in TLS-deficient adults.

Supplementary Figure 7. The hypothalamic-pituitary-gonadotrophic axis is disrupted in TLS-deficient adults

(a) Representative images of H&E-stained testis sections from 6-12-month-old wildtype, *Pcna*$^{R/R}$, *Rev1*$^{-/-}$ and *Rev7*$^{-/-}$ mice

(b) Quantification of interstitial cells, mostly Leydig cells (Data represent mean and s.d., P value calculated by two-tailed Mann-Whitney *U*-test; n=4 independent mice per genotype except for *Rev7*$^{-/-}$ n=3).

(c) Representative H&E-stained sections of ovaries from 3-, 6- and 12-month-old wildtype and *Pcna*$^{R/R}$ mice (similar results were obtained from 3 independent animals per genotype).

(d) Quantification of ovary mass from 3-, 6- and 12-month-old wildtype and *Pcna*$^{R/R}$ mice (Data represent mean and s.d., P value calculated by two-tailed Mann-Whitney *U*-test; n=18, 2, 10, 5, 6, and 13 independent animals, left to right).

(e) Quantification of ovarian mass in 2-4- and 6–12-month-old *Pcna*$^{R/R}$, *Rev1*$^{-/-}$ and *Rev7*$^{-/-}$ mice (Data represent mean and s.d.; 2-4 months, n=3, 2 and 4, 6-12 months, n=12, 2 and 2 independent animals for *Pcna*$^{R/R}$, *Rev1*$^{-/-}$ and *Rev7*$^{-/-}$ respectively).

(f) Quantification of serum luteinizing hormone concentrations in male and female wildtype and *Pcna*$^{R/R}$ mice (Data represent mean and s.d., P value calculated by two-tailed Mann-Whitney *U*-test; n=5, 4, 14 and 13 independent animals, left to right).

(g) Quantification of serum follicle stimulating hormone (FSH) concentration in male and female wildtype and *Pcna*$^{R/R}$ mice (Data represent mean and s.d., P value calculated by two-tailed Mann-Whitney *U*-test; n=4, 4, 14 and 13 independent animals, left to right).

(h) Representative H&E-stained ovaries from 12-month-old *Pcna*$^{R/R}$ mice demonstrating pathological changes of ovarian stromal hyperplasia, including overspill of hyperplastic stromal cells into the adjacent fat at the hilum (left), surface epithelial inclusions (center) and areas of hyperthecosis (right).

(i) Pathological assessment of stromal cell hyperthecosis of ovaries from 6-12-month-old wildtype, *Pcna*$^{R/R}$, *Rev1*$^{-/-}$ and *Rev7*$^{-/-}$ mice (Data represent mean and s.d.; n=14, 13, 2 and 2 independent animals, left to right).

(j) Representative images of H&E-stained ovary sections from 6-12-month-old wildtype, *Pcna*$^{R/R}$, *Rev1*$^{-/-}$ and *Rev7*$^{-/-}$ mice and the assessment of stromal hyperplasia (Data represent mean and s.d.; n=14, 13, 2 and 2 independent animals, left to right).

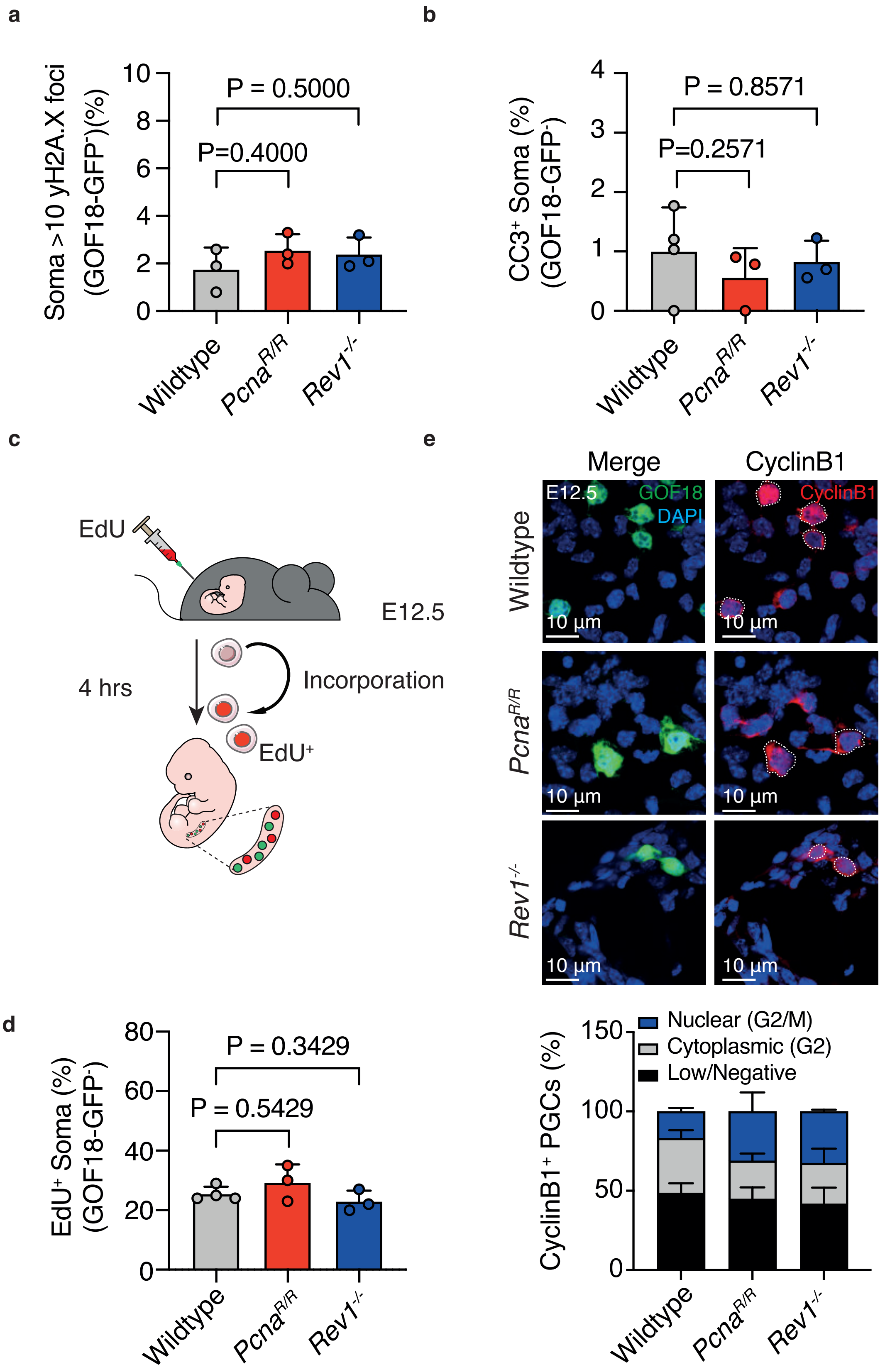

Supplementary Figure 8. PCNA K164 and REV1 are dispensable in gonadal somatic cells.

Supplementary Figure 8. PCNA K164 and REV1 are dispensable in gonadal somatic cells

(a) Frequency of somatic cells (GFP^-) with >10 ɣ-H2A.X foci per nucleus from wildtype, *Pcna*$^{R/R}$ and *Rev1*$^{-/-}$ embryos at E12.5 (Data represent mean and s.d., P value calculated by two-tailed Mann-Whitney *U*-test; n=3 for all genotypes).

(b) Quantification of the frequency of cleaved-caspase 3 (CC3)-positive somatic cells from wildtype, *Pcna*$^{R/R}$ and *Rev1*$^{-/-}$ embryos at E12.5 (Data represent mean and s.d., P value calculated by two-tailed Mann-Whitney *U*-test; n=4, 3 and 3, left to right).

(c) Schematic of EdU pulse experiment. Pregnant dams were injected with EdU and culled 4 hrs later to harvest E12.5 embryos. The genital ridges of these embryos were analyzed for EdU incorporation using IF.

(d) Quantification of the frequency of EdU^+ somatic cells of the genital ridge from wildtype, *Pcna*$^{R/R}$ and *Rev1*$^{-/-}$ embryos at E12.5 following a 4-hour EdU pulse (Data represent mean and s.d., P value calculated by two-tailed Mann-Whitney *U*-test; n=3 per genotype).

(e) Top: Representative images of E12.5 gonads stained for cyclin B1 and GFP. Bottom: Frequency of PGCs with nuclear, cytoplasmic or low/negative cyclin B1 staining (Data represent mean and s.d.; n=5, 3 and 3, left to right).

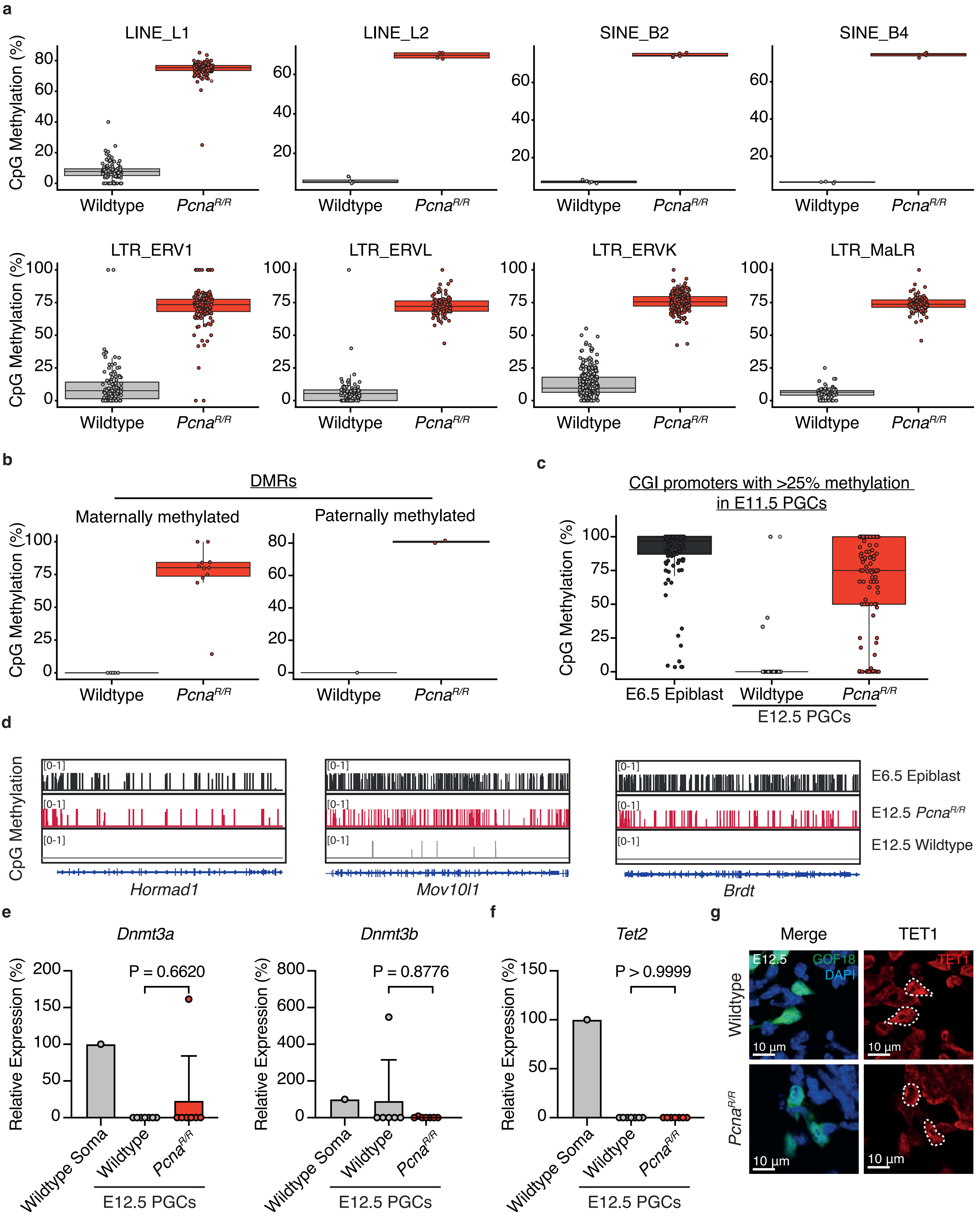

Supplementary Figure 9. Global retention of methylation in E12.5 *Pcna*$^{R/R}$ PGCs.

Supplementary Figure 9. Global retention of methylation in E12.5 *Pcna*$^{R/R}$ PGCs

(a) Quantification of DNA CpG methylation across repeat elements in the genomes of wildtype E12.5 wildtype and *Pcna*$^{R/R}$ PGCs. The center of boxplots displays the median, boxes display the interquartile range and the whiskers show the minimum and maximum of percentage methylation calculated over each feature with each point representing an individual feature (LINE_L1 n= 130, 132; LINE_L2 n= 4, 4; SINE_B2 n= 5, 5; SINE_B4 n= 4, 4; LTR_ERV1 n= 117, 142; LTR_ERVL n= 105, 109; LTR_ERVK n= 277, 277; LTR_MaLR n= 102, 108; left to right).

(b) Quantification of DNA CpG methylation across maternally and paternally demethylated DMRs in the genomes of E12.5 wildtype and *Pcna*$^{R/R}$ PGCs. Quantification of DNA CpG methylation across repeat elements in the genomes of wildtype E12.5 wildtype and *Pcna*$^{R/R}$ PGCs. The center of boxplots displays the median, boxes display the interquartile range and the whiskers show the minimum and maximum of percentage methylation calculated over each locus with each point representing an individual locus (Maternally methylated DMR n= 5, 10; Paternally methylated DMR n= 1, 2; left to right).

(c) Quantification of DNA CpG methylation in wildtype E6.5 epiblast cells and E12.5 wildtype PGCs and *Pcna*$^{R/R}$ PGCs across CGI-containing promoters which show >25% methylation at E11.5. The center of boxplots displays the median, boxes display the interquartile range and the whiskers show the minimum and maximum of percentage methylation calculated over each promotor with each point representing an individual promotor (n=123, 52, 181 left to right).

(d) IGV visualization of CpG methylation across selected GRR genes in E6.5 epiblast cells from wildtype embryos, E12.5 wildtype PGCs and E12.5 *Pcna*$^{R/R}$ PGCs. The plots represent the distribution of CpG methylation across genes segmented in 0.1 Kbp genomic windows.

(e) RT-qPCR expression analysis of *Dnmt3a* and *Dnmt3b* in the spleen of wildtype mice (wildtype soma) and PGCs from wildtype and *Pcna*$^{R/R}$ E12.5 embryos. (Data represent mean and s.d., P value calculated by two-tailed Mann-Whitney *U*-test; for *Dnmt3a*, n=1, 7 and 7 left to right; for *Dnmt3b*, n=1, 6 and 7 left to right).

(f) RT-qPCR expression analysis of *Tet2* in the spleen of wildtype mice (wildtype soma) and PGCs from wildtype and *Pcna*$^{R/R}$ E12.5 embryos. (Data represent mean and s.d., P value calculated by two-tailed Mann-Whitney *U*-test; n=1, 7 and 7 left to right).

(g) Representative images of E12.5 gonads stained for TET1 and GFP.

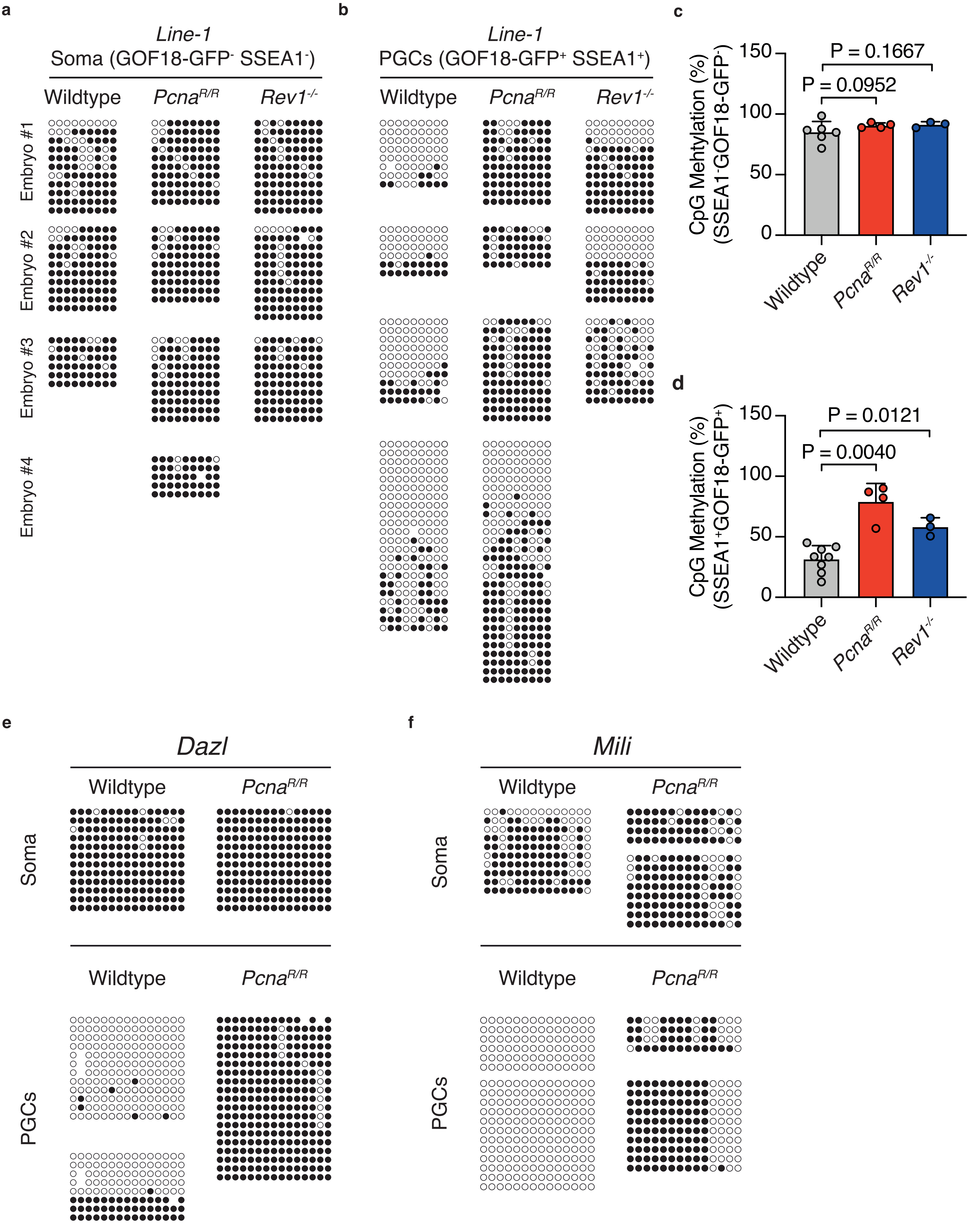

Supplementary Figure 10. *Pcna*$^{R/R}$ and *Rev1*$^{-/-}$ PGCs retain DNA methylation.

Supplementary Figure 10. *Pcna*$^{R/R}$ and *Rev1*$^{-/-}$ PGCs retain DNA methylation

(a) Genomic bisulfite sequencing reads of the CpG-rich region of the *Line-1* element from FACS-purified somatic cells from E12.5 wildtype, *Pcna*$^{R/R}$ and *Rev1*$^{-/-}$ embryos (filled = methylated CpG, open = unmethylated CpG).
(b) Genomic bisulfite sequencing reads of the CpG-rich region of the *Line-1* element from FACS-purified PGCs from E12.5 wildtype, *Pcna*$^{R/R}$ and *Rev1*$^{-/-}$ embryos
(c) Quantification of methylated CpG dinucleotides in the *Line-1* element in somatic cells from E12.5 wildtype, *Pcna*$^{R/R}$ and *Rev1*$^{-/-}$ embryos (Data represent mean and s.d., P value calculated by two-tailed Mann-Whitney *U*-test; n=3, 4 and 3 left to right).
(d) Quantification of methylated CpG dinucleotides in the *Line-1* element in PGCs from E12.5 wildtype, *Pcna*$^{R/R}$ and *Rev1*$^{-/-}$ embryos (Data represent mean and s.d., P value calculated by two-tailed Mann-Whitney *U*-test; n=4, 4 and 3 left to right).
(e) Genomic bisulfite sequencing reads of the CpG-rich region of the *Dazl* gene promoter element from FACS-purified PGCs from E12.5 wildtype and *Pcna*$^{R/R}$ embryos.
(f) Genomic bisulfite sequencing reads of the CpG-rich region of the *Mili* gene promoter element from FACS-purified PGCs from E12.5 wildtype and *Pcna*$^{R/R}$ embryos.

a

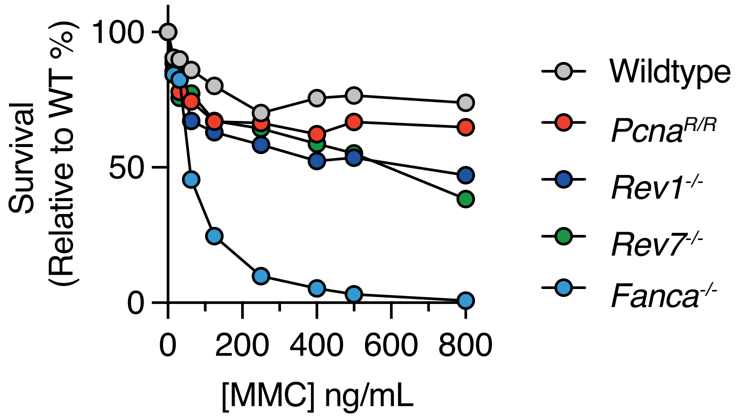

Supplementary Figure 11. Sensitivity of TLS- and FA-deficient cells to MMC.

Supplementary Figure 11. Sensitivity of TLS- and FA-deficient cells to MMC.

Supplementary Data Figure 1. Sensitivity of TLS- and FA-deficient cells to MMC.

(**a**) Sensitivity of wildtype, *Pcna*$^{R/R}$, *Rev1*$^{-/-}$, *Rev7*$^{-/-}$ and *Fanca*$^{-/-}$ cells to MMC (data represent 3 independent experiments each carried out in triplicate).

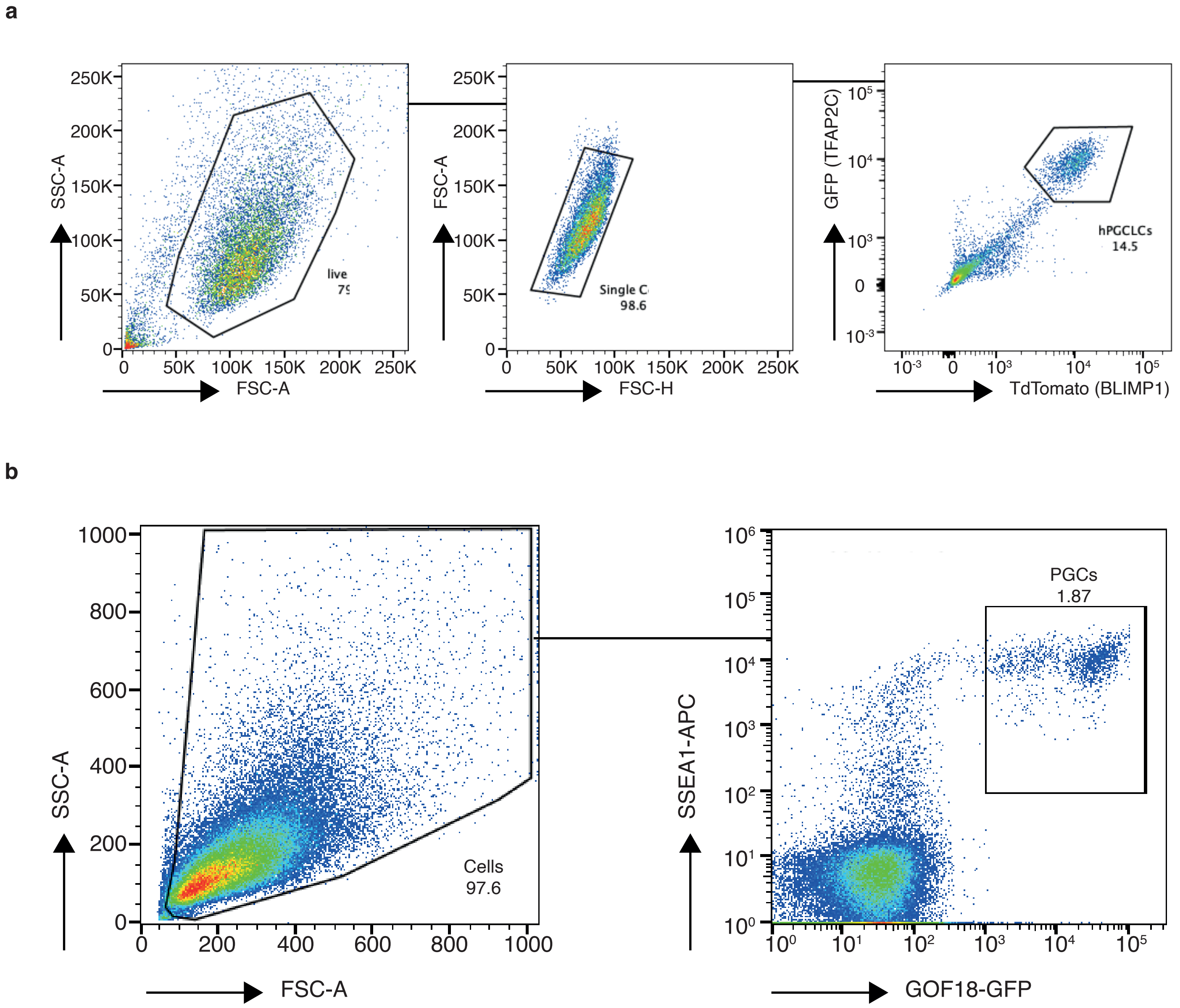

Supplementary Figure 12. Flow cytometry gating strategies for hPGCLCs and mPGCs

Supplementary Figure 12.

(a) Gating strategies for quantification of hPGCLCs.
(b) Gating strategies for quantification of PGCs from mouse embryos.

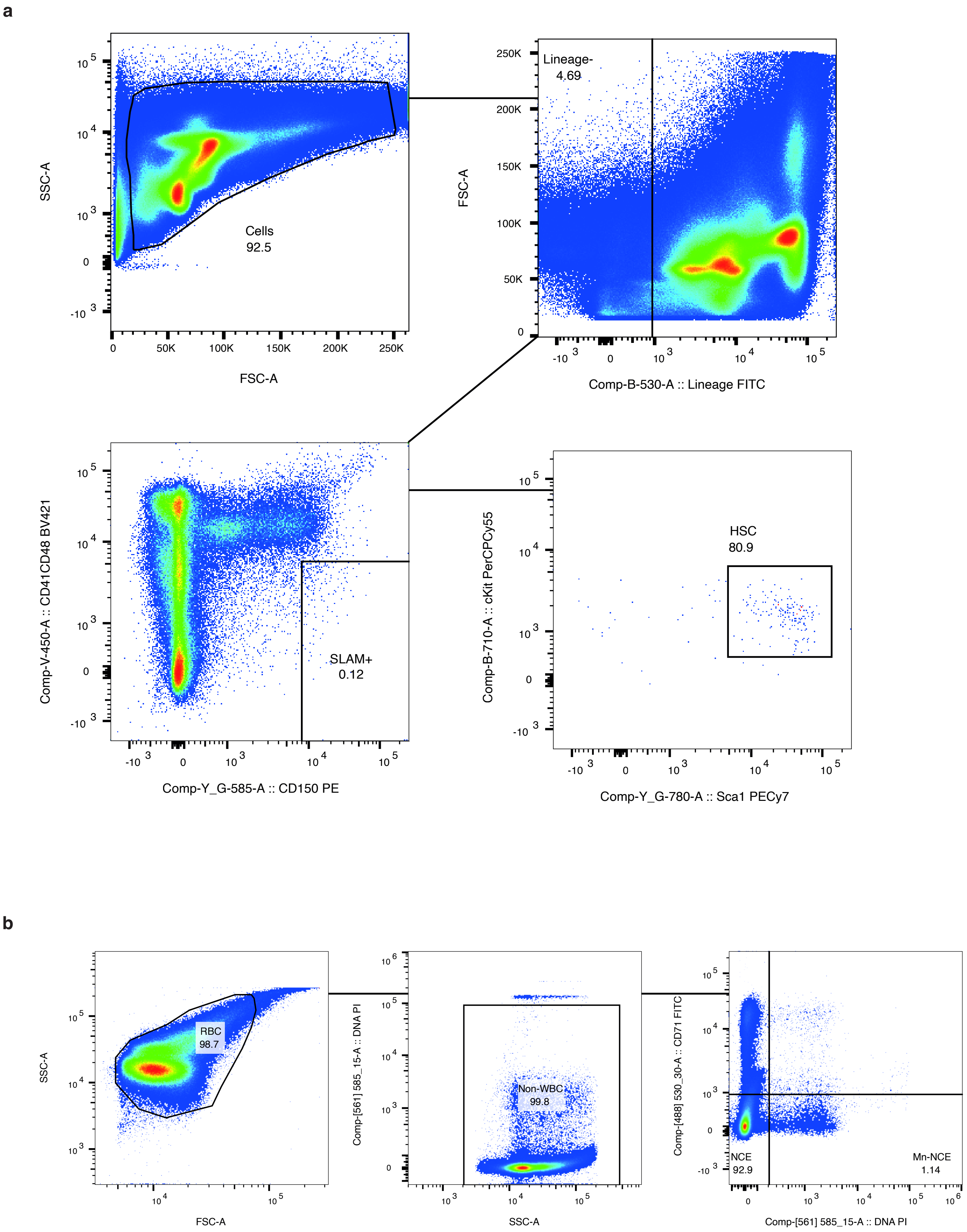

Supplementary Figure 13. Flow cytometry gating strategies for HSC and Micronucleus assay

Supplementary Figure 13.
(a) Gating strategies for quantification of HSCs.
(b) Gating strategies for quantification of Mn-NCEs.

a

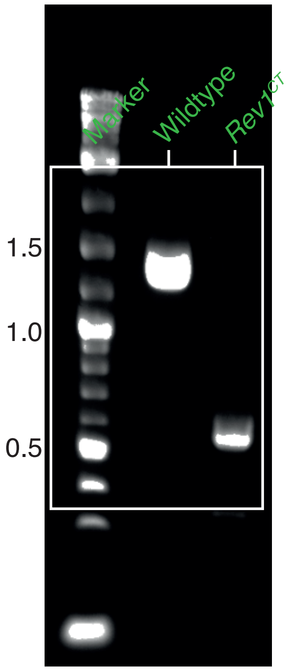

Supplementary Figure 2c

b

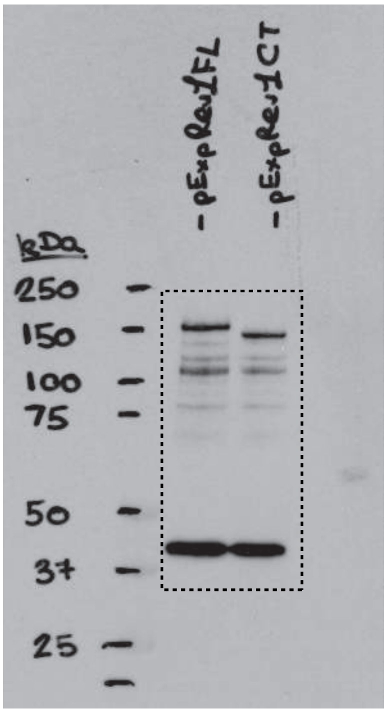

Supplementary Figure 2d

Supplementary Figure 14. Uncropped gels and western blots.

Supplementary Figure 14.

(a) Uncropped western blot from Supplementary Fig. 2c.
(b) Uncropped western blot from Supplementary Fig. 2d.

Supplementary Tables:

Supp. Table 1	Genotyping
PcnaK164R	Custom Taqman
Rev1AA	Taqman
GOF18-GFP & Stella-GFP-1	AGTGCTTCAGCCGCTACC
GOF18-GFP & Stella-GFP-2	GAAGATGGTGCGCTCCTG
GOF18-GFP & Stella-GFP-Pr	FAM-TTCAAGTCCGCCATGCCCGAA-TAMRA
Rev1_1	ATTGTGAGTCTCTAGCGTTTG
Rev1_2	GCTGGAATTGAAATTCTAGG
Rev1_3	GCTTCCATTGCTCAGCGGTG
Rev1CT_1	GTTGTACAGCTGAGCTCGGA
Rev1CT_2	TACCTCACAAGCACTGCTGG
Rev1CT_3	AGCAGTCGGTGGAGTCTGTA
PolQ_1	TGCAGTGTACAGATGTTACTTTT
PolQ_2	TGGAGGTAGCATTTCTTCTC
PolQ_3	TCACTAGGTTGGGGTTCTC
PolQ_4	CATCAGAAGCTGACTCTAGAG

PolK_1	CTGATGTGACCGCTGTTAAATGTTG
PolK_2	CTGTGGAGATGCCTTAGCGG
PolK_3	GATCCTGCAATCAATAGCTCACGG
Rev7_1	TCCAGGACACACTCCACTGC
Rev7_2	CGTTCTGCAAGCACAGGAAC
Rev7_3	TCGTGGTATCGTTATGCGCC

Supplementary Table 1. Assays used to genotype mice.

Supp. Table 2	CRISPR
hREV1 gRNA1	TTCCTCCAATTTCTGGACCT
hREV1 gRNA2	ATCAGATGCTGCTATGCAGA
hREV1-screen-F	TGGTCACTAGCACAGAATAAGGT
hREV1-screen-R	ACATGCCAAAATAGGGTTAAGTAAA
Pcna Donor	GAACAGGAGTACAGCTGTGTAATAAAGATGCCGTCGGGTGAATTTGCACGTATATGCCGAGACCTTAGCCACATTGGAGATGCTGTTGTGATATCCTGTGCAAGGAACGGTGTGAAGTTTTCTGCAAGTGGAGAGCTTGGCAATGGGAACATTAAGTTGTCACAAACAAGTAATGTGGATAAAGAAGAGGAGGCG
Pcna sgRNA	TGATATCCTGTGCAAAGAAT
Rev1AA Donor	TTTGGTCTTGTCTTTGATTTCAATACGGAGGGCAGCTGCAAACTCCTCAGGAGAAAGTTTCGTCTCTGCAAGGATGTCCGTGACGTCAATCAGTGCTGCCGCGCAGCTGACAGCTTCGATGCTGTGTGTGTAGCTATCAGGACAAAGAGAAGGCAGTGAGTGAGTCACACCTAGGGGCGGCTCTGGGAGTTGTCAGATAC
Rev1AA sgRNA	CCTCGATGCTGTGTGTGTAGCTA
Rev1CT Donor	CAAAATACTGACTGTTACTAGATTTTAATGTACTTGACACTGTCTGAATACAGCATTCTCGTTCACTTCCTCAACAGGAAGAAGTCTCGGCTTCTACCCTAATGGTTTTACAGCAGACTTATGGAAGCACACTGAAAGTGACCTGACTGCTGTGCAGAGGGCCTGGGGCTCTCTGCGCTGTGCCAGCAGTGCTTGTGAGG
Rev1CT sgRNA1	AATACGACCAGAATGGATTG
Rev1CT sgRNA2	TAAGGCACCATTTGAACTAT
Rev1CT-Seq-F	GTTGTACAGCTGAGCTCGGA
Rev1CT-Seq-R	TACCTCACAAGCACTGCTGG

Supplementary Table 2: Oligonucleotide sequences for CRISPR editing and validation.

**Supp. Table 3**	**qPCR**
Gapdh	NM_008084.2
Ddx4	Mm00802445_m1
Nanos3	Mm00808138_m1
Prdm1	Mm01187285_m1
Polq	Mm00712819_m1
Rev1	Mm0045983_m1
Polk	Mm01282564_m1
Rev7	Mm00510936_m1
Rev3l	Mm01181854_m1
Stella_F	GCTAACCCTAAACCCCGGTGT
Stella_R	CAATGCGGTTCCGTAGACTGC
Fragilis_F	CAGCACCTTGGTCCTCAGCA
Fragilis_R	CAGGACCGGAAGTCGGAATC
Dazl_F	TCTTTGCCAGATATGGCTCAGT
Dazl_R	CTTCTGCACATCCACGTCATTA
Mili_F	TTGGCCTCAAGCTCCTAGAC
Mili_R	GAACATGGACACCAAACCTACA
Sycp3_F	GCAGTCTAGAATTGTTCAGAGCCAGA
Sycp3_R	TCCAAACTCTTTATGAACTGCTCGTG

Mael_F	TGGCCACTCTCTTTGGAATC
Mael_R	GCATTTCCAATTCTTCCAGC
Mov10l1_F	CGCTGTGACGAGTACAGTG
Mov10l1_R	CTGACAACCCTTTGCTAGAGTTT
Hormad1_F	GGCTCCTAGCTGTTTCAGTATCT
Hormad1_R	TTGTCCCATAAGCACGTTCTG
Brdt_F	AGTGGGCGGTTGACGAATC
Brdt_R	AGTCAGGCAGCTTTAGTTTCAC
Tet1_F	GAGCCTGTTCCTCGATGTGG
Tet1_R	CAAACCCACCTGAGGCTGTT
Tet2_F	TGTTGTTGTCAGGGTGAGAATC
Tet2_R	TCTTGCTTCTGGCAAACTTACA
Dnmt1_F	AAGAATGGTGTTGTCTACCGAC
Dnmt1_R	CATCCAGGTTGCTCCCCTTG
Dnmt3a_F	CGACCCATGCCAAGACTCACCTTCCAG
Dnmt3a_R	AGACTCTCCAGAGGCCTGGT
Dnmt3b_F	ACTGCCTGGAGTTCAGTAGGA
Dnmt3b_R	CCCTGTCTGATGGAGTTCGAC
Gata4_F	CTAGACCGTGGGTTTTGCAT
Gata4_R	TGGGTTAAGTGCCCCTGTAG
Gata6_F	ACCACCTTATGGCGCAGAAA

Gata6_R	ATAGCAAGTGGTCTGGGCAC
Pax6_F	GTCCATCTTTGCTTGGGAAA
Pax6_R	TAGCCAGGTTGCGAAGAACT
Nestin_F	GCTCAGGTCCTGGAAGGTC
Nestin_R	TAAGAAAGGCTGGCACAGGT
Sox1_F	GAAATAGCCAATGCCAGGTG
Sox1_R	CCGTGAATACGATGAGTGTTACC
Eomes_F	AAGGGGAGAGTTTCATCATCCC
Eomes_R	GGCGCAAGAAGAGGATGAAATAG
RPL0_F	GAAACTCTGCATTCTCGCTTCC
RPL0_R	ACTCGTTTGTACCCGTTGATGA

Supplementary Table 3: Assays used for gene expression analyses.
