# Supplementary material for: Primordial germ cell DNA demethylation and development require DNA translesion synthesis
Source: Nat Commun. 2024 May 3;15:3734. doi: 10.1038/s41467-024-47219-2 (PMC11068800; doi:10.1038/s41467-024-47219-2)
Supplement: Supplementary file 3 — Reporting Summary [file 41467_2024_47219_MOESM3_ESM.pdf]

nature portfolio

Corresponding author(s): Crossan
Last updated by author(s): Feb 14, 2024

Reporting Summary

Nature Portfolio wishes to improve the reproducibility of the work that we publish. This form provides structure for consistency and transparency in reporting. For further information on Nature Portfolio policies, see our Editorial Policies and the Editorial Policy Checklist.

Statistics

For all statistical analyses, confirm that the following items are present in the figure legend, table legend, main text, or Methods section.

n/a	Confirmed	
☐	☒	The exact sample size (*n*) for each experimental group/condition, given as a discrete number and unit of measurement
☐	☒	A statement on whether measurements were taken from distinct samples or whether the same sample was measured repeatedly
☐	☒	The statistical test(s) used AND whether they are one- or two-sided<br>*Only common tests should be described solely by name; describe more complex techniques in the Methods section.*
☒	☐	A description of all covariates tested
☒	☐	A description of any assumptions or corrections, such as tests of normality and adjustment for multiple comparisons
☐	☒	A full description of the statistical parameters including central tendency (e.g. means) or other basic estimates (e.g. regression coefficient) AND variation (e.g. standard deviation) or associated estimates of uncertainty (e.g. confidence intervals)
☒	☐	For null hypothesis testing, the test statistic (e.g. *F*, *t*, *r*) with confidence intervals, effect sizes, degrees of freedom and *P* value noted<br>*Give P values as exact values whenever suitable.*
☒	☐	For Bayesian analysis, information on the choice of priors and Markov chain Monte Carlo settings
☒	☐	For hierarchical and complex designs, identification of the appropriate level for tests and full reporting of outcomes
☒	☐	Estimates of effect sizes (e.g. Cohen's *d*, Pearson's *r*), indicating how they were calculated

Our web collection on statistics for biologists contains articles on many of the points above.

Software and code

Policy information about availability of computer code

Data collection	WGBS sequencing was performed on an Illumina NextSeq 500 instrument. Flow cytometry in FACSDivav6.5 (BD) .
Data analysis	Bismark v0.23.1 was used to align DNA reads to the bisulfite converted GRCm38 mouse genome. For analysis of genomic features (e.g. promoters, gene bodies, 5Kbp tiles), CpG methylation rates were computed assuming a binomial model as previously (https://doi.org/10.1038/s41586-019-1825-8). To generate the Circos plot, the pycircos library was used. Flow cytometry was on FlowJo v10 (FlowJo LLC). Data was analysed in GraphPad Prism. Immunofluorescence was analysed in Fiji.

For manuscripts utilizing custom algorithms or software that are central to the research but not yet described in published literature, software must be made available to editors and reviewers. We strongly encourage code deposition in a community repository (e.g. GitHub). See the Nature Portfolio guidelines for submitting code & software for further information.

Data

Policy information about availability of data

All manuscripts must include a data availability statement. This statement should provide the following information, where applicable:

- Accession codes, unique identifiers, or web links for publicly available datasets
- A description of any restrictions on data availability
- For clinical datasets or third party data, please ensure that the statement adheres to our policy

The BS-Seq data generated in this study has been deposited in the Gene Expression Omnibus database under accession code GSE253991 [https://

www.ncbi.nlm.nih.gov/geo/query/acc.cgi?acc=GSE253991]. The BS-Seq data for E6.5 epiblast used in this study has been previously deposited in the Gene Expression Omnibus database under accession code GSE121708 [http://www.ncbi.nlm.nih.gov/geo/query/acc.cgi?acc=GSE121708]. Source data has been provided with this paper as a Source Data File. All materials are available on request from the corresponding authors.

Research involving human participants, their data, or biological material

Policy information about studies with human participants or human data. See also policy information about sex, gender (identity/presentation), and sexual orientation and race, ethnicity and racism.

Reporting on sex and gender	N/A
Reporting on race, ethnicity, or other socially relevant groupings	N/A
Population characteristics	N/A
Recruitment	N.A
Ethics oversight	N.A

Note that full information on the approval of the study protocol must also be provided in the manuscript.

Field-specific reporting

Please select the one below that is the best fit for your research. If you are not sure, read the appropriate sections before making your selection.

☒ Life sciences ☐ Behavioural & social sciences ☐ Ecological, evolutionary & environmental sciences

For a reference copy of the document with all sections, see nature.com/documents/nr-reporting-summary-flat.pdf

Life sciences study design

All studies must disclose on these points even when the disclosure is negative.

Sample size	For the analysis of Mendelian segregation of alleles, samples size was determined by power analysis using the following site http://biomath.info/power/chsq1gp.htm. Sufficient animals were used in order to detect a 50% reduction in expected frequency, using power of 0.8 and alpha 0.05. Others samples sizes were based on previously published results :PGC quantification PMID:31367016,25010009 ; hPGCLC quantification PMID:21820164; DNA damage assessment PMID:31367016,9488723.
Data exclusions	No data were excluded from analyses.
Replication	All experiments were repeated as described in the figure legends or materials and methods and for each experiment all attempts of replication were successful. Different biological replicates were performed on separated days, with samples collected from separated cell culture batches or independent biological samples collected from different animals on different days and processed on different days. Samples were allocated to groups based upon subsequent genotyping. Experiments were reproducible across all repeats. All replication data are included and confirm the original findings.
Randomization	No randomization was involved as groups are based upon the genotype of the animal/emrbyo or cell line.
Blinding	The investigators were blind to the genotypes of mice and relied solely on identification numbers throughout the study. For cellular assays the investigators were not blinded.

Reporting for specific materials, systems and methods

We require information from authors about some types of materials, experimental systems and methods used in many studies. Here, indicate whether each material, system or method listed is relevant to your study. If you are not sure if a list item applies to your research, read the appropriate section before selecting a response.

Materials & experimental systems

n/a	Involved in the study
☐	☒ Antibodies
☐	☒ Eukaryotic cell lines
☒	☐ Palaeontology and archaeology
☐	☒ Animals and other organisms
☒	☐ Clinical data
☒	☐ Dual use research of concern
☒	☐ Plants

Methods

n/a	Involved in the study
☒	☐ ChIP-seq
☐	☒ Flow cytometry
☒	☐ MRI-based neuroimaging

Antibodies

Antibodies used

anti-OCT4 (1:200, catalog no. ab181557; Abcam); anti-TFAP2C (1:100, catalog no. sc-12762; Santa Cruz); anti-SOX17 (1:200, catalog no. AF1924; R&D); Donkey anti-rabbit lgG (Alexa Fluor 594, catalog no. A21207; lnvitrogen); Donkey Anti-Goat lgG H&L (Alexa Fluor 488, catalog no. ab150129; Abeam); Donkey anti-Mouse lgG (H&L) (Alexa fluor 594, catalog no. R37115; Life Technologies); anti-PLZF (1:200, sc-28319) and anti-Ki67 (1:200, ab16667); swine anti-rabbit (1:200, catalog no. P0339, Dako) or anti-mouse horseradish peroxidase (HRP)-conjugated immunoglobulins (1:200, catalog no. P0339, Dako); anti-GFP (1:500, catalog no. GF090RI; Nacalai); anti-GFP (1:2,000, catalog no. ab13970); anti-phospho-Histone H2A.X (Ser139) (1:1,000, catalog no. 05-636; Millipore); anti-cleaved caspase 3 (1:400, catalog no. 9661; Cell Signaling Technology); anti-MVH (ab27591); anti-RPA32 (1:100, catalog no, 2208; Cell Signalling Technology); anti-cyclin B1 (1:100, catalog no. 4138; Cell Signalling Technology); anti-phospho-Chk2 (Thr68) (1:100, catalog no, 2661; Cell Signalling Technology); anti-TET1 (1:100, ab272901); anti-phospho-histone 3 (1:200, catalog no, 9701; Cell Signalling Technology); goat anti-rat Alexa Fluor 488 (1:1000, catalog no. A11006, Thermo Fisher Scientific), goat anti-chicken Alexa Fluor 488 (1:1000, catalog no. A11039, Thermo Fisher Scientific), goat anti-mouse Alexa Fluor 594 (1:1000, catalog no. All032, Thermo Fisher Scientific), goat anti-rabbit Alexa Fluor 594 (1:1000, catalog no. A21429; Thermo Fisher Scientific), goat anti-rat Alexa Fluor 594 (1:1000, catalog no. A21209; Thermo Fisher Scientific); anti-SSEA1 conjugated to Alexa Fluor 647 (1:100, catalog no. MC-480; Biolegend); anti-CD4 (1:100 clone H129.19, BD Pharmingen), CD3e (1:100 clone 145-2Cll, eBioscience), Ly-6G/Gr-1 (1:100 clone RB6-8C5, eBioscience), CDllb/Mac-1 (clone Ml/70, BD Pharmingen), CD45R/B220 (clone RA3-6B2, BD Pharmingen), Feε Rl α (clone MAR-1, eBioscience), CD8a (1:100 clone 53-6.7, BD Pharmingen), CDllc (1:100 clone N418, eBioscience); TER-119 (1:100 clone Ter119, BD Pharmingen), anti-c-Kit (1:100 PerCP- Cy5.5, clone 2B8, eBioscience), anti-Sca-1 (1:100 PE-Cy7, clone D7, eBioscience); anti-CD48 (1:100 FITC, clone HM48-1, BioLegend), anti-CD41 (FITC, clone MWReg30, BD Pharmigen), anti-CD150 (APC, clone TC15-12F12.2, BioLegend); anti-CD45R/B220 (1:100 PE, clone RA3-6B2, BD Pharmingen); anti-lgM (1:100 APC, clone 11/41, BD Pharmingen); anti-TER-119 (1:100 APC, clone Ter-119, BD Pharmingen); anti-CD71 (PE, clone C2, BD Pharmingen); anti-CDllb/Mac-1(APC, clone Ml/70, BD Pharmingen); anti-Ly-6G/Gr-1(1:100 PE, clone RB6-8C5, eBioscience); CD4 (1:100 FITC, clone H129.19, BD Pharmingen); CD8a (1:100 PE, clone 53-6.7, BD Pharmingen); anti-FLAG (1:200, M2 clone, catalog no. F1804, Sigma-Aldrich); anti-β-actin (1:1000, catalog no. ab8227; Abcam); swine anti-rabbit lg HRP-conjugated (1:3000, catalog no. P0399, Dako); goat anti-mouse lg HRP-conjugated (1:5000, catalog no. P0447, Dako); FITC-conjugated CD71 antibody (1:100 GenTex, clone Rl 7217.1.4).

Validation

Antibodies were validated by the manufacturer or in previous publications as indicated below:

Oct4: https://www.abcam.com/products/primary-antibodies/oct4-antibody-epr17929-chip-grade-ab181557.html

TFAP2C: https://www.scbt.com/p/ap-2gamma-antibody-6e4-4

SOX17: https://www.rndsystems.com/products/human-sox17-antibody_af1924

PLZF: https://www.scbt.com/p/plzf-antibody-d-9, PMID:36919611

ki67: https://www.abcam.com/products/primary-antibodies/ki67-antibody-sp6-ab16667.html

GFP: https://www.nacalaiusa.com/products/view/101/anti-gfp-rat-igg2a-monoclonal-gf090r

phospho-Histone H2A.X (Ser139) :https://www.merckmillipore.com/GB/en/product/Anti-phospho-Histone-H2A.X-Ser139-Antibody-clone-JBW301,MM_NF-05-636

cleaved caspase 3: https://www.cellsignal.com/products/primary-antibodies/cleaved-caspase-3-asp175-antibody/9661

MVH: https://www.abcam.com/products/primary-antibodies/ddx4--mvh-antibody-mabcam27591-ab27591.html

RPA32: https://www.cellsignal.com/products/primary-antibodies/rpa32-rpa2-4e4-rat-mab/2208

Tet1: https://www.abcam.com/products/primary-antibodies/tet1-antibody-ab272901.html

Cyclin B1: https://www.cellsignal.com/products/primary-antibodies/cyclin-b1-antibody/4138

phospho-Chk2:https://www.cellsignal.com/products/primary-antibodies/phospho-chk2-thr68-antibody/2661

phospho-histone 3: https://www.cellsignal.com/products/primary-antibodies/phospho-histone-h3-ser10-antibody/9701

SSEA1: https://www.biolegend.com/fr-lu/products/alexa-fluor-647-anti-mouse-human-cd15-ssea-1-antibody-4819?

GroupID=GROUP20

CD4, CD3e, Ly-6G/Gr-1, CDllb/Mac-1, CD45R/B220, Feε Rl α, CD8a, CDllc ; TER-119, c-Kit, Sca-1 CD48, CD41, CD150: This panel has previously been validated PMID: 22922648, 25707806, 23827712, 15989959

FLAG: https://www.sigmaaldrich.com/GB/en/product/sigma/F1804

β-actin: https://www.abcam.com/products/primary-antibodies/beta-actin-antibody-ab8227.html

Eukaryotic cell lines

Policy information about cell lines and Sex and Gender in Research

Cell line source(s)	The hiPSC cell line (BTAG 585bl-868) was a kind gift from Prof. Mitinori Saitou (Institute for the Advanced Study of Human Biology, Kyoto, Japan).
Authentication	No authentication was undertaken
Mycoplasma contamination	Cells were mycoplasma free
Commonly misidentified lines (See ICLAC register)	No commonly misidentified lines were used in this study

Animals and other research organisms

Policy information about studies involving animals; ARRIVE guidelines recommended for reporting animal research, and Sex and Gender in Research

Laboratory animals	All mice were maintained on an isogenic C57BL/6J background. The Stella-GFP (Tg(Dppa3/EGFP)6-25Masu) allele (MGI ID: 5519126) was a kind gift from Azim Surani. B6;129P2-Polktm1.1Rsky/J allele (MGI ID: 2445458), B6.Cg-Polqtm1Jcs/J allele (MGI ID: 2155399), GOF18-GFP(Tg(Pou5f1-EGFP)2Mnn) (MGI ID: 3057158) JAX (stock ID: 004654) mice were purchased from The Jackson Laboratory. We imported the previously described C57BL/6NTac-Mad2l2<tm1a(EUCOMM)Wtsi>/WtsiCnbc strain (EM: 05374), frozen sperm from The European Mouse Mutant Archive and used to derive live mice.
Wild animals	No wild animals were used
Reporting on sex	For primordial germ cell analyses performed at embryonic stages of development, these were done before sexually divergent development of the germline occurs and hence data from both sexes was combined. Embryonic data from each sex was disaggregated and analyzed separately in Supplementary Fig. 4e which confirmed lack of sex-specific phenotypes. The sex of embryos was assigned using endpoint PCR. For analysis of adult reproductive tissues, males and females were treated separately as detailed in the text with sex assignment performed by investigators.
Field-collected samples	N/A
Ethics oversight	All animal experiments performed in this study were approved by the Medical Research Council's Laboratory of Molecular Biology animal welfare and ethical review body and conform to the UK Home Office Animal (Scientific Procedures) Act 1986 (License no PP6752216).

Note that full information on the approval of the study protocol must also be provided in the manuscript.

Plants

Seed stocks	N/A
Novel plant genotypes	N/A
Authentication	N/A

Flow Cytometry

Plots

Confirm that:

☒ The axis labels state the marker and fluorochrome used (e.g. CD4-FITC).

☒ The axis scales are clearly visible. Include numbers along axes only for bottom left plot of group (a 'group' is an analysis of identical markers).

☒ All plots are contour plots with outliers or pseudocolor plots.

☒ A numerical value for number of cells or percentage (with statistics) is provided.

Methodology

Sample preparation	Human PGCLC: Day 4 aggregates were collected and dissociated with 0.25% trypsin-EDTA (Life Technologies, 25200056) for 10 min at 37C under agitation. Cells were washed with PBS containing fetal bovine serum (FBS) and 0.1% bovine serum albumin (BSA) before being subjected to centrifugation. Dissociated cells were then resuspended in FACS buffer (PBS, 0.1% BSA) and filtered by a cell strainer (BD Biosciences).<br><br>Mouse PGCs: the entire embryo (E8.5-10.5) or the developing urogenital ridges (E11.5-13.5) were isolated from embryos and placed into 500 µL or 150 µL pre-warmed trypsin solution (2.5 µg.mL-1 trypsin (Gibco), 25 mM Tris, 120 mM NaCl, 25 mM KCl, 25 mM KH2PO4, 25 mM glucose, 25 mM EDTA, pH 7.6) respectively, and incubated at 37°C for 10 min. Subsequently, 1 µL of Benzonase endonuclease (Millipore) was added and the sample gently disaggregated by pipetting and incubated for 5 min at 37°C. The trypsin was inactivated by adding 1 mL of PBS/5% v/v fetal bovine serum and centrifuged at 3,300 rpm for 10 min. The sample was resuspended in 100 µL of anti-SSEA1 conjugated to Alexa Fluor 647 (catalog no. MC-480; Biolegend) diluted 1:100 in PBS/2.5% v/v fetal bovine serum and incubated for 10 min at room temperature. Samples were diluted by adding 300 µL of PBS/2.5% v/v fetal bovine serum and passed through a 70 µm filter.<br><br>Hematopoiesis Analysis: Bone marrow cells were isolated from the femora and tibiae of mutant mice and appropriate controls by flushing cells and passing them through a 70-µm filter. The following antibodies were used to stain for HSCs: FITC-conjugated lineage cocktail with antibodies anti-CD4 (clone H129.19, BD Pharmingen), CD3e (clone 145-2C11, eBioscience), Ly-6G/Gr-1 (clone RB6-8C5, eBioscience), CD11b/Mac-1 (clone M1/70, BD Pharmingen), CD45R/B220 (clone RA3-6B2, BD Pharmingen), Fcε R1α (clone MAR-1, eBioscience), CD8a (clone 53-6.7, BD Pharmingen), CD11c (clone N418, eBioscience) and TER-119 (clone Ter119, BD Pharmingen), anti-c-Kit (PerCP-Cy5.5, clone 2B8, eBioscience), anti-Sca-1 (PE-Cy7, clone D7, eBioscience). When staining for SLAM markers the same lineage cocktail was used (FITC) with the addition of the following antibodies: anti-CD48 (FITC, clone HM48-1, BioLegend), anti-CD41 (FITC, clone MWReg30, BD Pharmigen), anti-CD150 (APC, clone TC15-12F12.2, BioLegend) and anti-c-Kit and Sca-1 as above. Maturation of B cells was assessed using anti-CD45R/B220 (PE, clone RA3-6B2, BD Pharmingen) and anti-IgM (APC, clone II/41, BD Pharmingen). The maturation of the erythroid lineage was analysed using antibodies anti-TER-119 (APC, clone Ter-119, BD Pharmingen) and anti-CD71 (PE, clone C2, BD Pharmingen). Granulocyte–macrophage maturation was assessed with antibodies anti-CD11b/Mac-1 (APC, clone M1/70, BD Pharmingen) and anti-Ly-6G/Gr-1 (PE, clone RB6-8C5, eBioscience). Thymic T-cell maturation was assessed using CD4 (FITC, clone H129.19, BD Pharmingen) and CD8a (PE, clone 53-6.7, BD Pharmingen) antibodies. The samples were incubated for 15 min at 4 °C in the dark with the exception of samples containing anti-CD34 (RAM34), which were incubated for 90 min. Samples were run on a LSRII flow cytometer (BD Pharmingen) and the data were analysed with FlowJo v10.<br><br>Micronucleus Assay: Mice were bled and 62 µl blood was mixed with 338 µl PBS supplemented with 1,000 U ml−1 of heparin (Calbiochem). 360 µl of blood suspension was then added to 3.6 ml of methanol at −80 °C and stored at −80 °C for at least 12 h. 1 ml of fixed blood cells was then washed with 6 ml of bicarbonate buffer (0.9% NaCl, 5.3 mM NaHCO3). The cells were resuspended in 150 µl of bicarbonate buffer and 20 µl of this suspension was used for subsequent staining. 72 µl of bicarbonate buffer, 1 µl of FITC-conjugated CD71 antibody (GenTex, clone R17217.1.4) and 7 µl RNase A (Sigma) were premixed and added to 20 µl of each cell suspension. The cells were stained at 4 °C for 45 min, followed by addition of 1 ml bicarbonate buffer and centrifugation. Finally, cell pellets were resuspended in 500 µl bicarbonate buffer supplemented with 5 µg ml−1 propidium iodide (Sigma). The samples were analysed immediately on an LSRII FACS analyser (BD) and the data analysed with FlowJo v10.
Instrument	hPGCLC quanitification: Aria III, BD Biosciences<br>mPGC quanitifcation: ECLIPSE analyzer (Sony Biotechnology)<br>Hematopoiesis Analysis and Micronucleus Assay: LSRII flow cytometer (BD Pharmingen)
Software	Data was processed using FlowJo version 10
Cell population abundance	N/A
Gating strategy	For hPGCLC quantification, cells were gated using FSC/SSC and hPGCLCs defined using eGFP and tdTomato reporters.<br>For mPGC quantification, cells were gated using FSC/SSC and PGCs defined as (APC-SSEA+GFP+).

☒ Tick this box to confirm that a figure exemplifying the gating strategy is provided in the Supplementary Information.
